# Heat-Killed *Enterococcus faecalis* EF-2001 Attenuate Lipid Accumulation in Diet-Induced Obese (DIO) Mice by Activating AMPK Signaling in Liver

**DOI:** 10.3390/foods11040575

**Published:** 2022-02-16

**Authors:** Meiqi Fan, Young-Jin Choi, Nishala Erandi Wedamulla, Yujiao Tang, Kwon Il Han, Ji-Young Hwang, Eun-Kyung Kim

**Affiliations:** 1 Division of Food Bioscience, College of Biomedical and Health Sciences, Konkuk University, Chungju 27478, Korea; fanmeiqi@kku.ac.kr; 2 Department of Food Science and Nutrition, College of Health Science, Dong-A University, Busan 49315, Korea; choijang11@kku.ac.kr (Y.-J.C.); nishala.erandi@yahoo.com (N.E.W.); 3 Center for Silver-Targeted Biomaterials, Brain Busan 21 Plus Program, Dong-A University, Busan 49315, Korea; 4 Department of Health Sciences, The Graduate School of Dong-A University, Busan 49315, Korea; 5 Department of Export Agriculture, Faculty of Animal Science and Export Agriculture, Uva Wellassa University, Badulla 90000, Sri Lanka; 6 School of Bio-Science and Food Engineering, Changchun University of Science and Technology, Changchun 130600, China; yuanxi00@126.com; 7 KoreaBerm Co., Ltd., Wonju 26362, Korea; kihan@berm.co.kr; 8 Department of Food Science & Technology, Dong-Eui University, Busan 47340, Korea; hjy@deu.ac.kr; 9 Center for Food & Bio Innovation, Dong-A University, Busan 49315, Korea

**Keywords:** EF-2001, *Enterococcus faecalis*, lipid accumulation, obese, liver damage

## Abstract

To explore the inhibitory mechanism of heat-killed *Enterococcus faecalis*, EF-2001 on hepatic lipid deposition, a diet-induced obese (DIO) animal model was established by high-fat diet (HFD). The DIO C57BL/6 mice were divided into four groups: the normal group without HFD (ND, *n* = 8), obesity group (HFD, *n* = 8), experimental group (HFD + EF-2001, 200 mg/kg, *n* = 8), and positive control group (HFD + Orlistat, 60 mg/kg, *n* = 8). After 4 weeks, liver and adipose tissue were fixed in 10% paraformaldehyde, followed by embedding in paraffin for tissue sectioning. The differences in body mass, body fat ratio, fatty cell area, and lipid profiling of the liver (TC, LDL, and HDL) were also determined. Moreover, Western blot was performed to analyze the expression of lipid accumulation-related proteins, including AMPK, PPARγ, SREBP-1, ACC, and FAS. Compared with the HFD group, the HFD + EF-2001 group exhibited decreased fat mass, liver index, adipocyte area, TC, and LDL, and an increased level of HDL. The results of liver hematoxylin and eosin (H&E), and oil red O staining showed that the mice in each intervention group were improved on hepatic lipid accumulation, and the mice in the HFD + EF-2001 group were the most similar to those in the normal group when compared with the HFD group. From the Western blot results, we proved that EF-2001 activated the AMPK signaling pathway. EF-2001 significantly upregulated the expressions of p-AMPK and p-ACC and downregulated PPARγ, SREBP-1, and FAS in murine liver. Taken together, these results suggest that EF-2001 decrease lipid accumulation in the DIO model mice through the AMPK pathway and ameliorate liver damage by HFD.

## 1. Introduction

With the increasing proportion of high-calorie diets in the dietary composition of the population, the rate of overweight and obesity is rising [1]. A long-term high-fat diet may cause the energy intake of the body to exceed its energy consumption, and the excess energy will be stored as body fat, eventually leading to obesity and lipid metabolism disorders [2]. Homeostatic regulation of lipid metabolism is fundamental to maintaining the basic functions of the body, and disorders of lipid metabolism play a significant role in the development of diabetes, obesity, fatty liver, cardiovascular disease, and abnormal cell proliferation [3,4,5]. As a result, the focus of modern society has shifted to explore potential mechanism to effectively prevent and improve disorders of lipid metabolism. Since non-alcoholic fatty liver disease (NAFLD) has been associated with obesity, chronic oxidative stress, dyslipidemia, and inflammation, NAFLD has been viewed as a hepatic manifestation of metabolic syndrome [6]. Clinically, there are no drugs acknowledged for the treatment of NAFLD [7]. However, there are some hepatoprotective, enzyme-lowering, and lipid-lowering drugs for symptomatic management [8,9]. However, most of these drugs are associated with adverse side effects. As a result, safer and healthier non-drug therapies have been proposed, including the use of probiotics [10,11]. *Enterococcus faecalis* has been reported to promote intestinal microbiota balance, alleviate metabolic syndrome, and modulate immunity, among other functions [12]. *E. faecalis* is also effective in treating hyperlipidemia, obesity, and fatty liver disease [13].

In recent studies, it has been demonstrated that *E. faecalis* are not only beneficial when they are alive but also beneficial when they are dead [14]. HFD-induced obesity in mice can be ameliorated by heat-treated *E. faecalis* [15]. However, strain specificity must be considered. Therefore, in the present study, we focused our on the possibility of heat-inactivated *E. faecalis* ameliorates hepatic lipid accumulation, as administered by EF-2001. A probiotic strain of *E. faecalis* EF-2001 was isolated from healthy human feces and characterized. It has been reported that EF-2001 possesses radioprotective, antitumor, anti-chronic enteritis, and anti-atopic dermatitis properties [15,16,17,18]. In this way, EF-2001 can be used without the risk of infection or antibiotic resistance.

EF-2001 is a widely used, safe, and well-tolerated probiotics and may be useful as an adjuvant therapy for the treatment of hepatic lipid accumulation [19]. To date, the type of effect that EF-2001 demonstrates on hepatic lipid metabolism and its dominant mechanism remains unclear. In this study, we examined the effects of EF-2001 on hepatic lipid accumulation in DIO mice and examined the effects of TG synthesis, catabolism, and the AMPK signaling pathway to provide a new theoretical basis for the treatment of disorders of hepatic lipid metabolism.

## 2. Materials and Methods

### 2.1. Heat-Killed Enterococcus faecalis (EF-2001)

EF-2001 (heat-killed *Enterococcus faecalis*, KoreaBerm, Wonju, Korea), a commercially available probiotic, prepared in lyophilized form, was originally isolated from healthy human feces. It contains 7.5 trillion colony-forming units of dried EF-2001 per 1 g prior to being heat-killed. As a heat-inactivated dried powder, these are heat-treated dead cells added to the fermented milk.

### 2.2. Experimental Animals, Diet, and Treatments

Four-week-old C57BL/6 male mice (NARA Biotech, Seoul, Korea) were randomly divided into a normal control group (normal diet (ND), 10% kJ fat content, *n* = 10), a high-fat model group (HFD, 60% kJ fat content, *n* = 30), and maintained obese mouse model for 8 weeks after 1 week of adaptation to the experimental environment. After 8 weeks, mice with 20% higher body weight than the control group were selected and randomly divided into three groups (*n* = 8): saline-treated HFD-fed mice, EF-2001 (HFD + EF; orally at a dose of 200 mg/kg/day), and orlistat (HFD + Orl; orally at a dose of 60 mg/kg/day). Both EF-2001 and Orlistat were dissolved in saline, and the same daily gavage volume was ensured for each group. All mice were fed normal water, and the HFD, HFD + EF, and HFD + Orl groups were given high-fat chow. The ND group was given normal chow. Gavage was continued for 4 weeks. The clinical dosage of EF-2001 is one pack at a time for adults (1.5 g each), one time a day. After conversion, the clinical dosage for adults is 25 mg/kg/day (the adult weight is considered 60 kg). The equivalent dose for mice is 12.3 times that of adults [20]. Therefore, each mouse is best given 307.5 mg/kg/day probiotics. With reference to previous animal experiments [16,17,18], we reduced the dose to 200 mg/kg/day. In the same way, the dose of orlistat in mice was calculated to be 60 mg/kg/day. Orlistat is among the few types of over-the-counter diet pills available worldwide, and its long-term application is considered to have no toxic side effects. Ye et al. identified that compared with placebo, orlistat caused a significant reduction in body weight in patients with NAFLD. Thus, orlistat has been used as a positive treatment for strength enhancement [21]. Mice were fasted overnight after 12 weeks and then executed over anesthesia [22]. Konkuk University’s Institutional Animal Care and Use Committee approved all experiments, and every effort was made to minimize suffering and the number of animals used in this study (KU18090).

Blood samples were obtained by cardiac puncture under anesthesia, and serum was separated by centrifugated at 3000 rpm (848× *g*) for 20 min and stored at −80 °C until assayed. Adipose tissues (epididymal) were weighed, and livers were collected, frozen in liquid nitrogen, and stored at −80 °C until further analysis. Pieces of epididymal adipose and liver were fixed in 10% formaldehyde for further histological analyses.

### 2.3. Body Composition Analysis

Dual-energy X-ray absorptiometry (DXA) was used to measure the body fat. After 4 weeks of treatment, DXA measurements were taken using a total-body scanner (InAlyzer dual X-ray absorptiometry, Medikors, Gyeonggi, Korea). Several DXA measurements were taken under anesthesia, with low energy and high energy, to divide the images into gram units of bone and tissue by separating the samples into fat and lean before analysis.

### 2.4. Biochemical Analysis

After collecting blood samples from the cardiac puncture, the serum was separated by centrifugated at 3000 rpm (848× *g*) for 20 min and stored at −80 °C until further analysis. The serum levels of alanine aminotransferase (ALT), alkaline phosphatase (ALP), and aspartate aminotransferase (AST) were tested using an automated analyzer (Abaxis VETSVAN VS2 Chemistry Analyzer, Union City, CA, USA). The liver total cholesterol (TC), low-density lipoprotein cholesterol (LDL), and high-density lipoprotein cholesterol (HDL) were measured with a rapid blood lipid analyzer (OSANG healthcare Lipid Pro, Anyang, Korea). ELISA kits (Merck, Darmstadt, Germany) were used to measure serum leptin and adiponectin.

### 2.5. Histological Analysis

Histological examination of the liver and epididymal adipose tissue was performed by dissecting them, buffering them with 10% neutral formalin, and embedding them in paraffin. H&E staining was applied to formalin-fixed and paraffin-embedded tissue blocks that were cut into 4-micron-thick sections. Tissue sections were examined under an optical microscope (Leica DMi1; Leica Microsystems, Solms, Germany) at 200× magnification, and fat cell size was determined. The frozen liver sections were fixed for 10 min in a 10% formaldehyde solution, followed by rinsing with running water. The samples were then soaked in an isopropyl alcohol solution for 20–30 s, stained for 15–20 min with Oil-Red O, and rinsed with distilled water. The specimen was stained with hematoxylin for 40 s, followed by 5 min of soaking in tap water. This work was carried out as previously described [22]. Gelatin was heated and cooled in a water bath to achieve binding and stabilization. The slices were viewed under a microscope at a magnification of 200×, and the size of the fat cells was measured.

### 2.6. mRNA Expression Analysis

A total RNA extract was obtained from mouse livers using the TRIzol method, and the concentration and purity of RNA were determined using a nucleic acid protein concentration meter (PhileKorea, Korea). The experimental method described previously was used [23]. The primer sequences for the target genes are shown in Table 1. The relative expression of the mRNA was calculated using the default settings: ΔCt(test) = Ct(target gene, test) − Ct(GAPDH, test); ΔCt(calibrator) = Ct(target gene, calibrator) − Ct(GAPDH, calibrator); ΔΔCt = ΔCt(test) − ΔCt(calibrator); Relative gene expression ratio = 2^−ΔΔC^^t^. GAPDH as the internal reference gene.

### 2.7. Protein Expression Analysis

The liver tissues were mixed with protein lysate (RIPA-protein phosphatase inhibitor 99:1), and the lysate products were centrifuged at 13,000 rpm for 15 min at 4 °C. Protein concentrations were determined using a bicinchoninic acid protein concentration assay kit according to the instructions given in the kit, and protein samples were prepared at a mass concentration of 2 µg/µL. A sodium dodecyl sulfate-polyacrylamide gel electrophoresis (SDS-PAGE) was used to separate proteins with different molecular masses. Western blot antibodies were diluted 1:2000 with TBST buffer. Anti-p-AMPK (#2535), anti-PPARγ (#2435) and anti-ACC (#3676) were purchased from Cell Signaling Technology (Danvers, MA, USA). Anti-AMPK (sc-25792), anti-SREBP-1c (sc-13551), anti-p-ACC (sc-271965), anti-FAS (sc-55580), and anti-β-actin (sc-1616) were purchased from Santa Cruz Biotechnology (Santa Cruz, CA, USA). In this study, protein bands were visualized through enhanced chemiluminescence using a chemiluminescent substrate chromogenic agent. This work was carried out as previously described [24]. Images were acquired using a gel imager, and bands were quantified using software such as ImageJ 1.8 software (National Institutes of Health, Bethesda, MD, USA).

### 2.8. Statistical Analysis

Three or more experiments are summarized in each result. Statistical evaluations are expressed as mean ± SEM. The data were analyzed by one-way ANOVA with the Tukey’s test using the GraphPad Prism program (Version 8.4; GraphPad Software, Inc., San Mateo, CA, USA). *p* value < 0.05 was considered significant.

## 3. Results

### 3.1. Effect of EF-2001 on Growth Performance of DIO Mice

**Figure 1:**
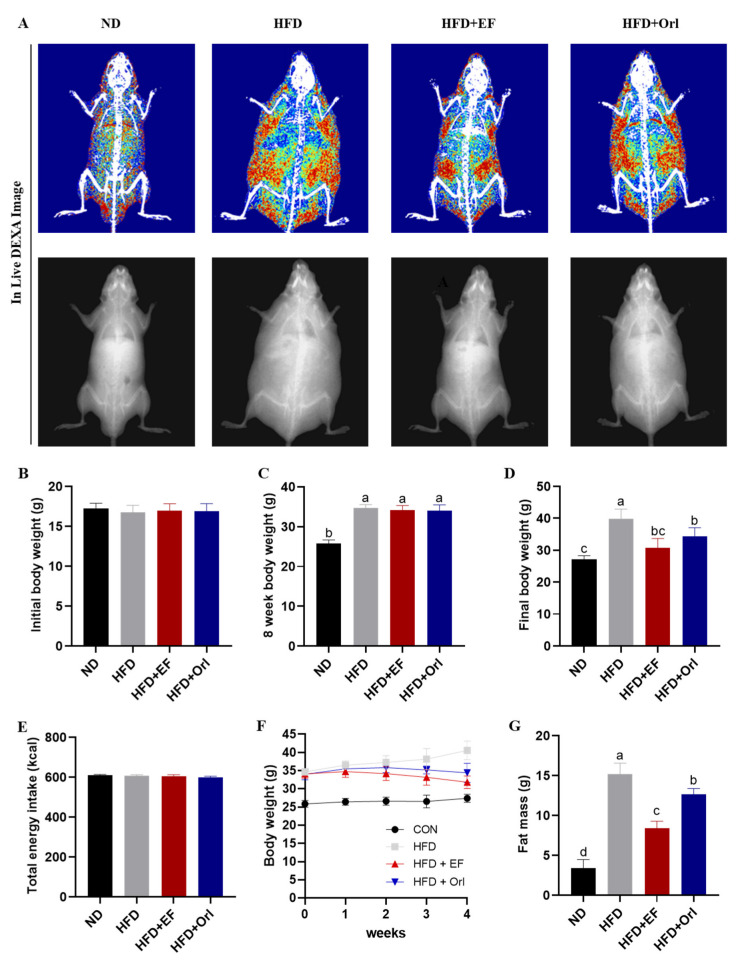
The effect of EF-2001 on growth performance of DIO mice. (**A**) The radiography of body fat. (**B**) Initial body weight. (**C**) Eight-week body weight. (**D**) Final body weight. (**E**) Total energy intake. (**F**) The trend of body weight change of mice in each group. (**G**) Fat mass. Dunnett’s multiple range tests revealed significant differences in ^a–d^ values with different superscripts at *p* < 0.05. Data are expressed as mean ± SEM.

**Figure 2:**
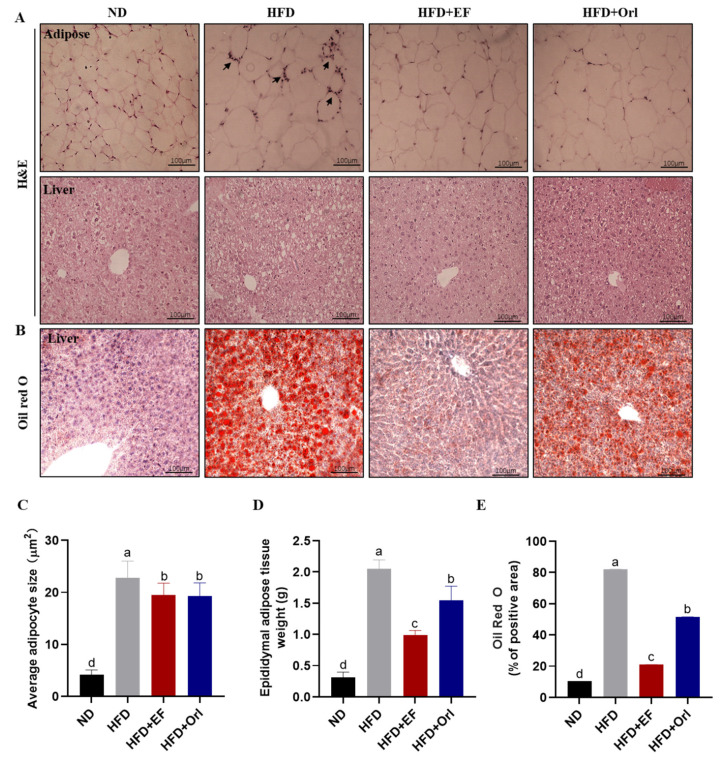
The effect of EF-2001 on liver and adipose histopathology. (**A**) Representative microscopic observation of adipose tissue of epididymis and liver tissue by H&E staining. Arrows mark the inflammatory cells. (**B**) Representative microscopic observation of liver tissue by oil red O staining. (**C**) Mean adipocyte area (μm^2^). (**D**) Epididymal adipose tissue weight (g). (**E**) Mean Oil Red O staining in hepatocytes area (μm^2^). Dunnett’s multiple range tests revealed significant differences in ^a–d^ values with different superscripts at *p* < 0.05. Data are expressed as mean ± SEM.

DXA was used to determine the effects of EF-2001 on fat mass. MCLW supplementation for 4 weeks significantly reduced the body mean area and fat mean area in DIO mice (Figure 1A). After 8 weeks, the body weight of HFD-fed mice was more than 20% higher than with the ND group. In the ND group, body weight increased by 8.28 ± 0.77 g. In the HFD-fed groups, body weight increased by 17.41 ± 1.67 g. It appears that the 8 weeks of high-fat diet feeding resulted in a significant increase in body weight compared to that of the ND group (*p* < 0.05). This indicates that the obese animal model was successfully modeled (Figure 1B,C). As shown in Figure 2E, there was no significant difference in energy intake between the groups during the 4-week experiment (*p* > 0.05). The results of the experiment were not affected by the amount of food intake. The ND and HFD groups continued to gain weight during the 4 weeks of the intervention, with Max values at week 4 of the experiment, 27.20 ± 1.06 g and 39.85 ± 2.99 g. The HFD + Orl group continued to gain weight during the first 2 weeks of the experiment, with Max values at week 3 of the experiment and a final weight of 34.41 ± 2.64 g. The HFD + EF group continued to gain weight during week 1 of the intervention, with Max values at week 1 of the experiment and a final weight of 34.41 ± 2.64 g. The HFD + EF group continued to grow in the first week of the intervention and gradually decreased in the following 3 weeks, with a final weight of 31.83 ± 1.75 g. (Figure 1D,F). On the other hand, the mean fat weight in the ND, HFD, HFD + EF, and HFD + Orl group was 3.37 ± 1.09 g, 15.17 ± 1.38 g, 8.36 ± 0.92 g, and 12.60 ± 0.76 g, respectively. Moreover, the EF-2001 intervention significantly reduced the fat mass due to high fat consumption (*p* < 0.05) (Figure 1G). Accordingly, the EF-2001 intervention significantly reduced body weight gain and fat mass levels in the HFD group compared with the ND group (Figure 1).

### 3.2. Effect of EF-2001 on Liver and Adipose Histopathology

Figure 2A (upper part) demonstrates the effect of EF-2001 on epididymal adipose tissue. Adipose tissue was observed microscopically after H&E staining. In the HFD group, the size of the adipocytes was significantly increased and accompanied by inflammatory cell infiltration compared with the ND group. The size of the adipocytes was significantly reduced by EF-2001 with orlistat intervention compared to the HFD group (Figure 2C), and the inflammatory response virtually disappeared. The mean adipose tissue weight of the epididymal in the ND, HFD, HFD + EF, and HFD + Orl group was 0.31 ± 0.09 g, 2.05 ± 0.14 g, 0.99 ± 0.08 g, and 1.55 ± 0.23 g, respectively (Figure 2D). EF-2001 significantly reduced adipose tissue weight in comparison to orlistat (*p* < 0.05). The effects of EF-2001 on liver pathology are illustrated in Figure 2A (lower part) and B. As a result of staining liver tissues with H&E and oil red O, the HFD group had an increased number of lipid droplets and balloon-like structures compared to the ND group (Figure 2A,B,E). In comparison to the HFD group, EF-2001 and orlistat intervention significantly reduced lipid accumulation in liver tissue and significantly alleviated hepatic steatosis. Moreover, EF-2001 treatment significantly decreased hepatic lipid accumulation compared to that of orlistat treatment (*p* < 0.05).

### 3.3. Effect of EF-2001 on Liver and Serum Biochemical Parameters

**Figure 3:**
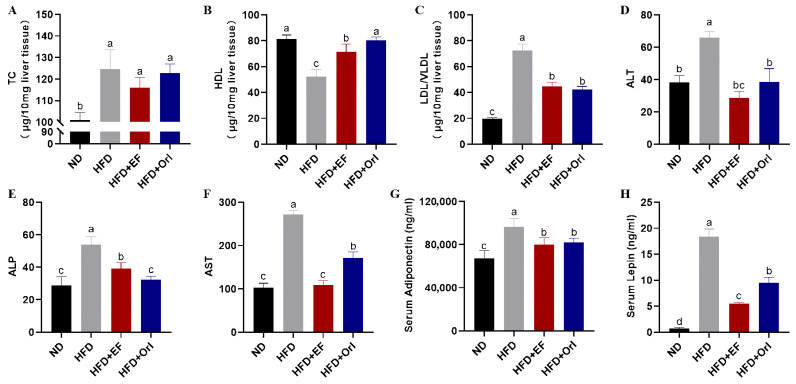
The effect of EF-2001 on liver and blood biochemical parameters. (**A**) Total cholesterol (TC) content of liver tissue. (**B**) High-density lipoproteins (HDL) content of liver tissue. (**C**) Low-density lipoproteins and very low-density lipoproteins (LDL/VLDL) content of liver tissue. (**D**) Serum alanine aminotransferase (ALT). (**E**) Serum alkaline phosphatase (ALP). (**F**) Serum aspartate aminotransferase (AST). (**G**) Serum adiponectin. (**H**) Serum leptin. Dunnett’s multiple range tests revealed significant differences in ^a–d^ values with different superscripts at *p* < 0.05. Data are expressed as mean ± SEM.

Figure 3A–C shows the effects of EF-2001 on liver biochemical indices. The TC level of the HFD group increased to 124.61 ± 8.94 μg/10 mg in comparison to that of the ND group (100.98 ± 3.41 μg/10 mg) (*p* < 0.05). After EF-2001 and orlistat intervention, the TC level decreased to 115.99 μg/10 mg and 122.76 μg/10 mg, respectively. However, there was no significant difference (*p* < 0.05) exhibited in the HFD + EF group and orlistat group compared to the HFD group (Figure 3A). HDL levels were significantly lower (*p* < 0.05) in the HFD group (52.31 ± 5.32 μg/10 mg) than that of the ND group (81.24 ± 3.23 μg/10 mg). EF-2001 significantly (*p* < 0.05) increased HDL (71.47 μg/10 mg) (Figure 3B). LDL/VLDL in the HFD group was 72.30 μg/10 mg, in contrast the ND group, which exhibited a significant decrease of 19.75 μg/10 mg (*p* < 0.05). Similarly, EF-2001 and orlistat displayed a significantly low (*p* < 0.05) LDL/VLDL value, 44.52 μg/10 mg and 42.45 μg/10 mg, respectively. Moreover, there were no significant difference in the LDL/VLDL values of the EF-2001 and orlistat interventions (Figure 3C).

Figure 3D–H illustrates the effect of EF-2001 on blood biochemical parameters. Compared with the ND group, the levels of ALT, ALP, and AST were significantly increased in the HFD group, whereas the levels of ALT, ALP, and AST were significantly decreased after EF-2001 and orlistat interventions (*p* < 0.05). Specifically, ALT and AST reached the level of ND group after EF-2001 intervention (Figure 3D–F). In comparison to the ND group, serum adiponectin and leptin levels were significantly increased in the HFD group. However, they were significantly decreased after EF-2001 and orlistat interventions (*p* < 0.05) (Figure 3G,H).

### 3.4. Effect of EF-2001 on Hepatic Lipid-Related Gene Expression

**Figure 4:**
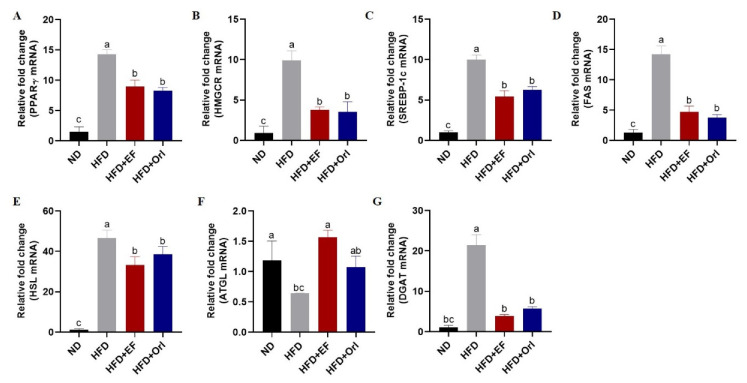
The effect of EF-2001 on hepatic lipid-related gene expression. mRNA expression in mouse liver as measured by real-time PCR. The charts showed expression levels with (**A**) PPARγ, (**B**) HMGCR, (**C**) SREBP-1c, (**D**) FAS, (**E**) HSL, (**F**) ATGL, and (**G**) DGAT. Dunnett’s multiple range tests revealed significant differences in ^a–c^ values with different superscripts at *p* < 0.05. Data are expressed as mean ± SEM.

Peroxisome proliferator-activated receptors (PPARγ), sterol regulatory element-binding protein 1 (SREBP-1c), 3-Hydroxy-3-Methylglutaryl-CoA Reductase (HMGCR), fatty acid synthase (FAS), hormone-sensitive triglyceride lipase (HSL), diacylglycerolacyl transferase (DGAT), and adipose triglyceride lipase (ATGL)are key substances involved in lipid metabolism. Based on Figure 4, liver lipid synthesis genes PPARγ, HMGCR, SREBP-1c, FAS, HSL, and DGAT mRNA were significantly higher in the HFD group compared with the ND group (*p* < 0.05), while fat oxidative catabolism genes ATGL mRNA were decreased (*p* < 0.05) (Figure 4). In response to the administration of EF-2001 and orlistat, liver liposynthesis-related genes (PPARγ, HMGCR, SREBP-1c, FAS, HSL, and DGAT) were significantly reduced in the HFD + EF and HFD + Orl group in comparison to the HFD group (*p* < 0.05) (Figure 4). In addition, the expression of ATGL mRNA, which is related to hepatic lipid oxidative catabolism, was significantly higher in the HFD + EF group compared to the HFD + Orl group (*p* < 0.05) (Figure 4). There was no significant difference in the regulation of lipid synthesis genes between the HFD + EF and HFD + Orl group in DIO mice. As a result, EF-2001 and orlistat can regulate the expression of genes related to lipid synthesis and oxidative catabolism in the liver, thus controlling the disorders of lipid metabolism caused by high-fat diets.

### 3.5. Effect of EF-2001 on the Expression of Hepatic Lipid-Related Proteins

**Figure 5:**
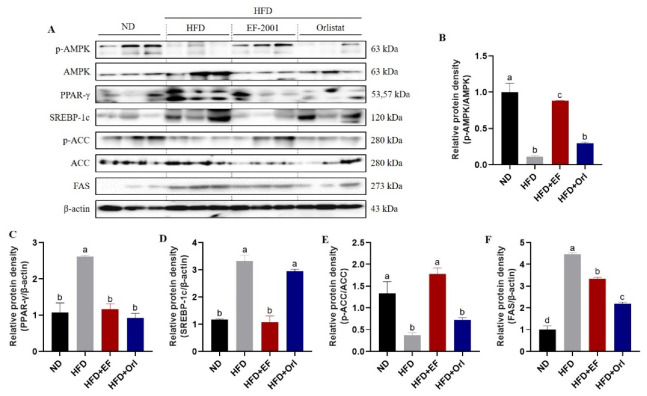
The effect of EF-2001 on the expression of hepatic lipid-related proteins. (**A**) Western blot. Normalized relative protein expression data. (**B**) p-AMPK/AMPK, (C) PPARγ/β-actin, (**D**) SREBP-1c/β-actin, (**E**) p-ACC/ACC, (**F**) FAS/β-actin. Dunnett’s multiple range tests revealed significant differences in ^a–d^ values with different superscripts at *p* < 0.05. Data are expressed as mean ± SEM.

The results are shown in Figure 5. The levels of phosphor-AMP-activated protein kinase (p-AMPK) and phospho-Acetyl-CoA carboxylase (p-ACC) were significantly reduced (*p* < 0.05), while the levels of AMP-activated protein kinase (AMPK), PPARγ, SREBP-1c, phospho-Acetyl-CoA carboxylase (ACC), and FAS were significantly increased (*p* < 0.05) in the livers of mice in the HFD group compared with the NC group. In conjunction with Figure 1, Figure 2, Figure 3 and Figure 4, this is further evidence that the significant weight gain of the organism is associated with disturbances in lipid metabolism caused by a high-fat diet. EF-2001 and orlistat treatment increased the expression levels of p-AMPK and p-ACC in livers of mice and decreased the expression levels of AMPK, PPARγ, SREBP-1c, ACC, and FAS (*p* < 0.05) (Figure 5). These results indicate that EF-2001 and orlistat can interfere with the high-fat diet-induced phosphorylation of hepatic AMPK and ACC in DIO mice. In addition, these compounds inhibited the maturation of AMPK, PPARγ, SREBP-1c, ACC, and FAS, and decreased the disturbance of lipid metabolism in the organism.

## 4. Discussion

Using EF-2001 and orlistat in high-fat diet-induced DIO mice, we found that the intervention alleviated hepatic lipid accumulation and steatosis. EF-2001 significantly reduced body weight, body weight gain, and fat mass, with greater efficacy than orlistat. In comparison with orlistat, EF-2001 had a more effective lipid-lowering effect. In addition, EF-2001 and orlistat significantly improved hepatic cholesterol levels [25]. There is an excessive accumulation of lipids in steatosis [26]. Moreover, hepatic lipid accumulation is the result of an imbalance between the acquisition and disposal of lipids. Orlistat and EF-2001 reduced liver TC levels, but there was no significant difference. There is a possibility that EF-2001 and orlistat increased HDL levels and decreased LDL/VLDL levels, resulting in no significant difference in TC. The combination of EF-2001 and orlistat resulted in significant reductions in serum ALT, ALP, and AST levels. Elevated serum levels of ALT, ALP, and AST have been reported to indicate liver damage, and ALT and AST are recognized as important markers for assessing liver injury [27,28,29]. In this study, EF-2001 was shown to reduce hepatic LDL levels and decrease hepatic lipid deposition in DIO mice, suggesting that it may be effective in reducing visceral and blood lipid levels in DIO mice. EF-2001 reduced body weight gain, fat weight, and lipid levels in the same manner as evident by previously reported studies in relation to *E. faecalis* [15].

Under normal physiological conditions, FFA is esterified to TG in the endoplasmic reticulum, resulting in lipid droplets that are stored in adipose tissues [30]. There is always an imbalance between energy absorption and energy expenditure, resulting in obesity. Excess energy is stored primarily as TGs [31]. TG can be deposited in non-adipose tissues when there is an excess of FFA in the body [32]. The liver maintains a relative balance between TG synthesis and catabolism [33]. To investigate the mechanism of lipid reduction in the liver, we examined the genes associated with lipid metabolism. DGAT is the primary rate-limiting enzyme in the TG synthesis process [34]. Studies have demonstrated that knockdown of DGAT can significantly reduce the accumulation of TG in cells [35]. In this experiment, EF-2001 significantly reduced the expression of DGAT in the liver of DIO mice, suggesting that EF-2001 could effectively inhibit lipid synthesis in the liver. Catabolism of TG plays a key role in maintaining hepatic TG homeostasis [36]. ATGL and HSL are the primary enzymes involved in TG catabolism, and they catalyze TG and DG catabolism, respectively [37]. Research has shown that the knockdown of ATGL in the liver causes a significant increase in hepatic lipid droplets and fatty liver in mice [38]. Conversely, knocking down the HSL gene resulted in a significant reduction in the TG levels in mice [39]. This experiment demonstrated that EF-2001 significantly promoted the expression of ATGL and inhibited the expression of HSL in the liver of DIO mice, indicating that EF-2001 promoted lipid catabolism. The results of this experiment revealed that EF-2001 significantly reduced lipid accumulation in the liver of DIO mice.

It is known that the accumulation of TG in hepatocytes may cause systemic disorders of lipid metabolism [36,40]. In contrast, AMPK is a key regulator of energy metabolism in mammalian cells and is crucial to maintaining energy homeostasis in cells [41]. There is evidence that this enzyme can be involved in glucose transport and metabolism, lipid metabolism, and almost all energy metabolic processes in cell growth, including protein synthesis and conversion, autophagy, apoptosis, and various cellular processes of endoplasmic reticulum stress [42,43,44]. This is the switch that controls the activation of the anabolic and catabolic pathways. In the process of lipid catabolism and synthesis, it can decrease lipid synthesis and increase lipid catabolism by regulating the expression of key genes [45,46,47]. AMPK-deficient mice were found to develop hepatic steatosis within 5 weeks of being fed a high-fat diet, whereas the normal group developed hepatic fat only after 12 weeks of feeding a high-fat diet [48]. As AMPK is activated, it activates fatty acid β-oxidation and inhibits adipogenesis [49]. ACC is AMPK’s first downstream target, as it is involved in the synthesis of malonyl coenzyme A [50,51]. It has been shown that AMPK inhibits ACC activity by phosphorylating it and, as a result, stimulates fatty acid oxidation and reduces fatty acid synthesis [52,53,54]. These results indicate that EF-2001 significantly promoted AMPK and ACC phosphorylation in the hepatocytes of DIO mice and alleviated the disorders of lipid metabolism caused by the high-fat diet by promoting de novo lipid formation and fatty acid oxidation. Furthermore, EF-2001 inhibited the expression of SREBP-1c, a lipogenic transcription factor that is abundant in mammalian livers [55]. Upon overexpression of SREBP-1c, the entry of fatty acids into hepatocytes triggers a new lipogenic process. SREBP-1c translocates to the nucleus and regulates the expression of downstream targets, such as FAS and ACC [56,57,58]. FAS is responsible for the final step in fatty acid biosynthesis and for liver fat metabolism [59,60]. AMPK regulates HMG-CoA reductase, which is responsible for cholesterol synthesis [61]. The nuclear receptor transcription factor family includes PPAR-γ, an important member of the lipid metabolism pathway [62]. EF-2001 significantly increased the expression of p-AMPK and p-ACC in the liver of DIO mice, while downregulating the expression of AMPK, PPAR, HMGCR, SREBP-1c, ACC, and FAS.

**Figure 6:**
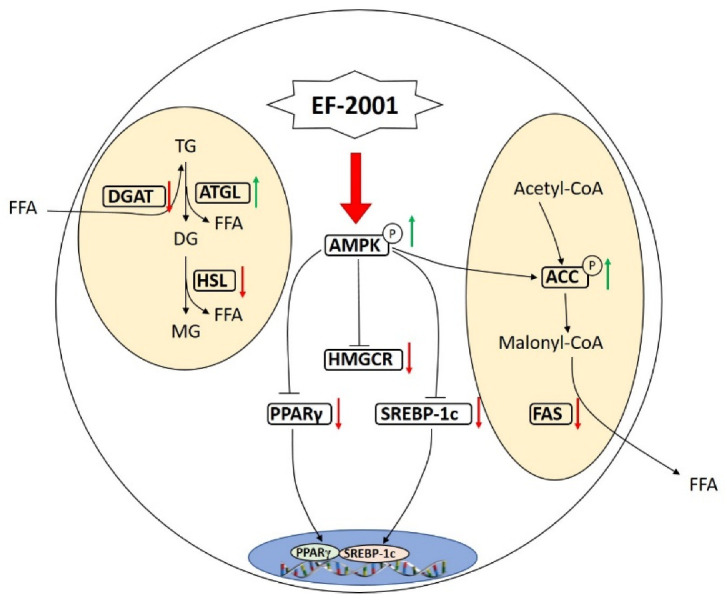
The mechanism of EF-2001 in alleviated hepatic lipid accumulation in DIO mice. EF-2001 promotes lipolysis by regulating AMPK and downstream signaling molecules. EF-2001 and these genes are linked by positive (green arrows) and negative (red arrows) feedback.

As a result, these findings suggest that EF-2001 regulates the lipid metabolism by mediating ACC phosphorylation through the AMPK pathway. Thus, EF-2001 is able to improve lipid metabolism disorder in DIO mice by inhibiting TG synthesis, promoting TG catabolism, and activating the AMPK signaling pathway. Figure 6 illustrates the mechanism by which EF-2001 could exert its hypolipidemic effects.

## Figures and Tables

**Table 1:** RT-PCR primers used in reverse transcription.

Target Genes	GenBank Accession	Primer Sequence	
PPAR-γ	NM_001308354.1	Forward	5-GAA AGA CAA CGG ACA AAT CAC-3
		Reverse	5-GAA ACT GGC ACC CTT GAA-3
HMGCR	NM_001360165.1	Forward	5-AGA ATA ATG TGC TAA GTA GTG CTA A-3
		Reverse	5-GCC TCT CTG AAC AAA GAC TC-3
SREBP-1C	NM_001358315.1	Forward	5-CTT CTG GAG ACA TCG CAA AC-3
		Reverse	5-GGT AGA CAA CAG CCG CAT C-3
FAS	NM_007988.3	Forward	5-CTT GGG TGC TGA CTA CAA CC-3
		Reverse	5-GCC CTC CCG TAC ACT CAC TC-3
HSL	NM_010719.5	Forward	5-AAG GAC TCA CCG CTG ACT TCC-3
		Reverse	5-GCC TGT CTC GTT GCG TTT GTA-3
ATGL	NM_025802.3	Forward	5-GAC CTG ATG ACC ACC CTT TCC-3
		Reverse	5-TGC TAC CCG TCT GCT CTT TCA-3
DGAT	NM_010046.3	Forward	5-CCT CAG CCT TCT TCC ATG AG-3
		Reverse	5-ACT GGG GCA TCG TAG TTG AG-3
GAPDH	NM_001289726.1	Forward	5-GCA CAG TCA AGG CCG AGA AT-3
		Reverse	5-GCC TTC TCC ATG GTG GTG AA-3

Peroxisome proliferator-activated receptors (PPAR-γ), 3-Hydroxy-3-Methylglutaryl-CoA Reductase (HMGCR), sterol regulatory element-binding protein 1 (SREBP-1C), fatty acid synthase (FAS), hormone-sensitive triglyceride lipase (HSL), adipose triglyceride lipase (ATGL), Diacylglycerolacyl transferase (DGAT), Glyceraldehyde-3-phosphate dehydrogenase (GAPDH).

## Data Availability

Not applicable.
